# Association between chronic kidney disease and cancer mortality: A report from the ALLHAT 

**DOI:** 10.5414/CN108949

**Published:** 2016-11-30

**Authors:** Dhruti P. Chen, Barry R. Davis, Lara M. Simpson, William C. Cushman, Jeffrey A. Cutler, Mirela Dobre, Charles E. Ford, Gail T. Louis, Paul Muntner, Suzanne Oparil, Linda B. Piller, Sara L. Pressel, Mark J. Sarnak, Paul K. Whelton, Jackson T. Wright, Mahboob Rahman

**Affiliations:** 1 Department of Medicine, Case Western Reserve University, University Hospitals Case Medical Center, Louis Stokes Cleveland Veterans Affairs Medical Center, Cleveland, OH,; 2 Coordinating Center for Clinical Trials, Division of Biostatistics, University of Texas School of Public Health, Houston, TX,; 3 Preventive Medicine Section, Memphis Veterans Affairs Medical Center, Memphis, TN,; 4 National Heart, Lung and Blood Institute, Bethesda, MD,; 5 Division of Nephrology and Hypertension, Case Western Reserve University, University Hospitals Case Medical Center, Louis Stokes Cleveland Veterans Affairs Medical Center, Cleveland, OH,; 6 Tulane University Health Sciences Center, New Orleans, LA,; 7 Department of Epidemiology at UAB,; 8 Department of Medicine, University of Alabama at Birmingham, Birmingham, AL, and; 9 Division of Nephrology, Tufts Medical Center, Boston, MA, USA; A list of the ALLHAT Collaborative Research Group members has been published previously, in JAMA. 2002; *288: *2981-2997.]

**Keywords:** chronic kidney disease, cancer, glomerular filtration rate

## Abstract

Background/objectives: Chronic kidney disease (CKD) and cancer are both common in older patients; whether CKD increases risk for cancer is unclear. This study evaluated CKD as a risk factor for cancer mortality in a large cohort of hypertensive patients. Study design: We did post-hoc analyses of in-trial and post-trial data from participants in the Antihypertensive and Lipid-Lowering Treatment to Prevent Heart Attack Trial (ALLHAT). Setting and participants: Participants were ≥ 55 years old with hypertension and one other additional risk factor for coronary heart disease. Predictor: Baseline estimated glomerular filtration rate (eGFR). Outcomes: Cancer mortality was ascertained by cancer-related deaths reported in national databases during and after the trial. Cox proportional hazard models were used to calculate hazard ratios (HRs) adjusted for possible confounders and were stratified by baseline GFR. Results: Participants’ mean age was 66.9 years. After a mean follow-up of 8.9 years, there were 2,338 reported cancer-related deaths. Participants with GFR < 45 mL/min/1.73 m^2^ were at increased risk of cancer mortality compared to those with GFR ≥ 90 mL/min/1.73 m^2^ (adjusted HR 1.54 (1.22 – 1.94), p-value for trend 0.004). These findings were consistent across subgroups defined by race, gender, and diabetes. Participants with GFR < 45 mL/min/1.73 m^2^ were at higher risk for mortality related to colon cancer (p-value for trend 0.048, HR 2.28 (1.12 – 4.62)) and urinary tract cancer (p-value for trend 0.001, adjusted HR 2.95 (1.14 – 7.65)). Limitations: This is a post hoc analysis of clinical trial data. Conclusions: In a large cohort of hypertensive patients, GFR < 45 mL/min/1.73 m^2^ was associated with a higher risk of cancer-related mortality.

Clinical trial registration: www.clinicaltrials.gov NCT 00000542 (registered October 27, 1999).

## Introduction 

It is estimated that more than 26 million Americans have chronic kidney disease (CKD). The burden of CKD is particularly high in the elderly: nearly one third of adults over the age of 70 have CKD [1]. While previous research evaluating the effect of CKD on clinical outcomes has focused on progression to end-stage kidney disease (ESRD) and cardiovascular disease, data are emerging that CKD has an impact on other important domains such as frailty, cognitive function, and quality of life [2, 3]. An outcome of particular importance in the aging population is cancer. Both CKD and cancer are common in older patients and carry considerable morbidity and health-care costs [2, 4]. Epidemiological studies suggest that ESRD patients and kidney-transplant recipients are at increased risk of cancer [5, 6, 7]. It would be important to determine whether CKD increases the risk of cancer; such an association would have considerable clinical, research, and public-health significance. 

Development of kidney disease among patients with cancer, in part related to nephrotoxic chemotherapy, is well described, but there have been conflicting data regarding CKD and its role in the development of cancer [2, 8, 9, 10, 11, 12, 14, 15]. Some, but not all, studies demonstrate an association between higher levels of urinary albumin-creatinine ratio and cancer incidence [16, 17, 18]. Recent studies have found cancer as a major source of death among those with CKD [13, 19]. This illustrates the increasing interest in the relationship between CKD and cancer, and additional epidemiologic studies are needed to further understand this association and set the stage for targeted pathophysiologic studies to understand the mechanisms of the association between CKD and cancer. 

The Antihypertensive and Lipid-Lowering Treatment to Prevent Heart Attack Trial (ALLHAT) randomized 42,418 hypertensive patients and ascertained long-term follow-up data in eligible study participants [20]. This cohort provides a unique opportunity to evaluate the relationship between CKD and cancer. The purpose of this paper was to evaluate the association between CKD and the development of cancer-related mortality in a large cohort of hypertensive subjects with and without CKD. 

## Methods 

The design and main results of the ALLHAT have been previously published [20, 21, 22]. In brief, the ALLHAT was designed to determine if occurrence of fatal coronary heart disease (CHD) or nonfatal myocardial infarction (MI) (the primary outcome) is lower for high-risk hypertensive patients, age ≥ 55, assigned to an angiotensin converting enzyme inhibitor (lisinopril), a calcium channel-blocker (amlodipine), or an alpha-blocker (doxazosin) compared with assignment to treatment with a thiazide-type diuretic (chlorthalidone). Participants were recruited between February 1994 and January 1998 at 623 sites in the USA, Canada, Puerto Rico, and US Virgin Islands. The trial was first registered with ClinicalTrials.gov as NCT 00000542 on October 27, 1999. All centers obtained institutional review board approval for the study, which was conducted in compliance with the Declaration of Helsinki, and all participants signed an informed consent form. 

Exclusion criteria included symptomatic heart failure, known left ventricular ejection fraction < 35% or serum creatinine > 2.0 mg/dL. Clinical data were collected during a baseline study visit by interview and questionnaire as well as laboratory tests that were performed at a single-central laboratory using standard assays. Serum creatinine level was measured using the VITROS chemistry system (Ortho-Clinical Diagnostics, Rochester, NY, USA) with a coefficient of variation of ~ 2%. The Modification of Diet in Renal Disease (MDRD) equation was used to estimate eGFR, the groups were stratified by CKD stages (stages 1, 2, 3A, 3B). Mean in-trial follow-up on active randomized therapy was 4.9 years. The doxazosin arm was stopped early due to futility for an effect on the primary end-point (CHD) and to increased cardiovascular disease (CVD), especially heart failure in the doxazosin compared with the chlorthalidone arm [22]. Due to their shorter period of follow-up, participants assigned to doxazosin were not included in the current analyses, consistent with other papers evaluating long-term follow-up of ALLHAT participants [21]. Following completion of the remaining arms in March 2002, extended follow-up of ALLHAT participants was accomplished using Social Security Administration and National Death Index databases for passive surveillance over an average of 4 years, providing an overall mean follow-up time of 8.9 years (4.9 years during the active treatment phase and 4 years following cessation of the trial) [20]. 

In our analyses, the endpoint was cancer mortality at any point after randomization into the ALLHAT using National Death Index diagnoses. During the in-trial phase, the investigators determined cause of death. The National Death Index (NDI) was used to identify all mortality data for the entire trial period, and death certificates were obtained for those deaths discovered solely through NDI to confirm patient identification. The NDI*Plus* provided ICD-10 codes with causes of death. Additional details of the ALLHAT mortality outcomes have been published elsewhere [21]. 

**Figure 1.**
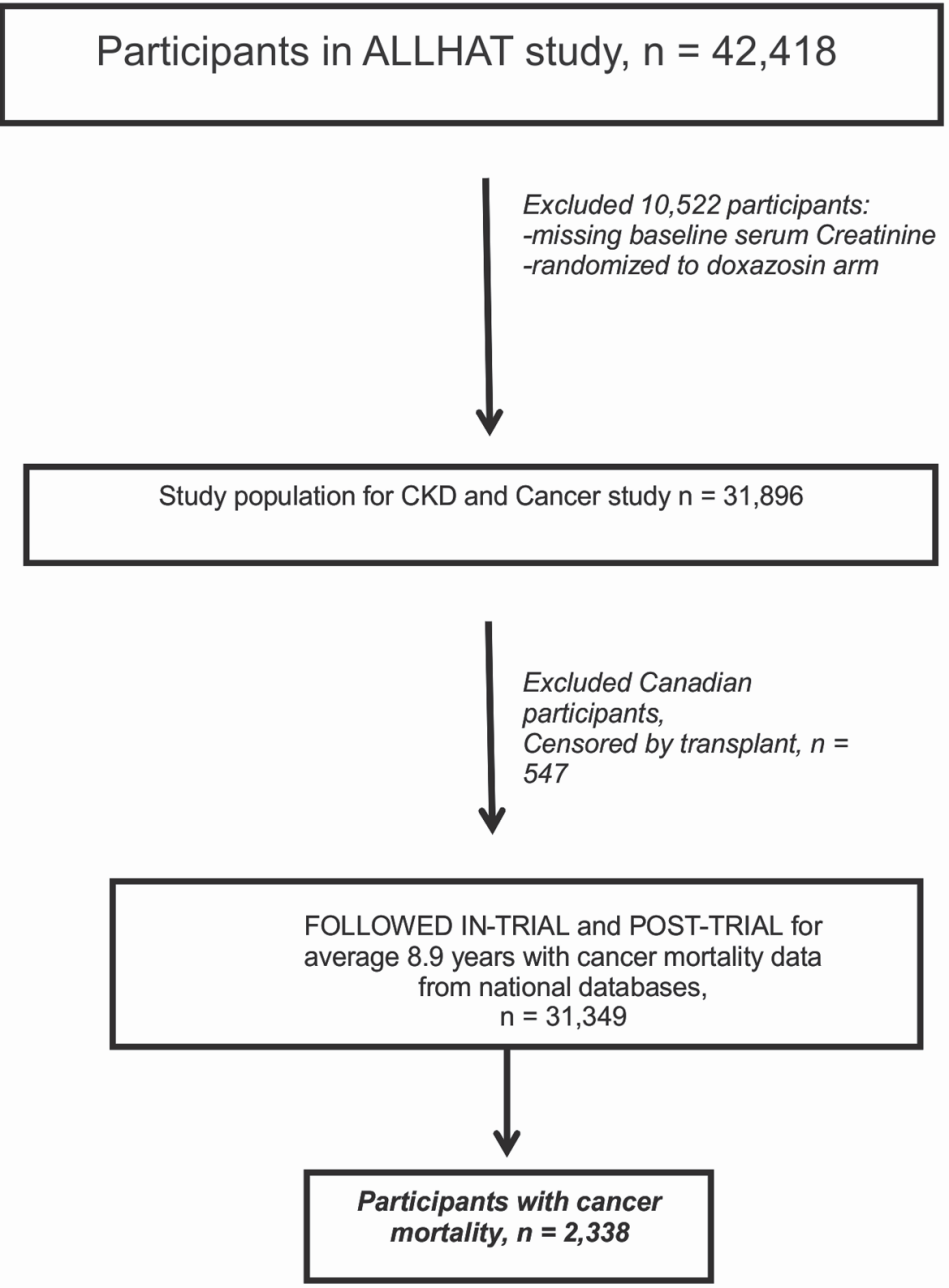
Study design.

Of the 42,418 participants enrolled in the ALLHAT, 10,522 were excluded from the analyses because they were assigned to doxazosin or had missing baseline serum creatinine data (Figure 1). The randomized groups were combined for the analyses reported here. The patients were further excluded if they were Canadian participants due to lack of access to long-term mortality data or censored by transplant. Cancer mortality data from 31,349 patients was analyzed. 

We also conducted another set of analyses with a composite endpoint of physician-reported cancer or cancer mortality during the in-trial (treatment) phase of the ALLHAT study. The ascertainment of nonfatal cancer incidence was accomplished by utilizing clinician reports with documentation, and cancer mortality was on physician reports or the National Death Index as described (also see Online Supplement) [21]. Investigator-reported fatal organ-specific cancers (categorical options, documented with pathology reports and/or death certificates) were mapped to International Classification of Disease-9 modification (ICD-9) for merging with post-trial data. If not identified by clinic investigator, cancer mortality was obtained from the National Death Index (NDI*Plus*) database. Deaths identified from 1999 forward were obtained in ICD-10 form and translated back to ICD-9. The organ-specific ICD-9 cancer mortality codes identified through NDI*Plus* were: prostate = 185, lung = 162, colon = 153 – 154.1, breast (in women) = 174, urinary tract = 188 – 189, hematopoietic = 200 – 208, and other = 141 – 199, 235 – 239. Detailed descriptions of the post-trial follow-up aims and procedures as well as the main results of the extended follow-up through 2006 have been published [21]. Deaths following the trial were ascertained using administrative databases [21]. Based upon physician report diagnoses or NDI*Plus* diagnoses for those lost to follow-up, cancer was classified by site of primary cancer, including prostate (in men), lung, colon, breast (in women), urinary tract (defined as kidney, ureter, ureterovesical junction, and bladder cancers), and “Other,” a consolidation of anus, bone, brain, esophagus, gallbladder/biliary, liver, malignant fibrous histiocytoma, melanoma, mesothelioma, neuroendocrine, oral cavity, ovary, pancreas, salivary, sinus, small intestine, sarcoma, squamous cell, stomach, testicular/penile/scrotal, tongue, throat, thyroid, uterus, vagina/vulva, and unknown or unspecified primary diagnoses. 

Baseline characteristics were compared across levels of eGFR using one-way analysis of variance for continuous covariates and contingency table analysis for categorical data. Cumulative event rates were calculated using the Kaplan-Meier method. Cox proportional hazards regression models and trend tests incorporated the participant’s entire trial experience to the end of the post-trial follow-up period. Participants who died during the follow-up period were censored at the time of death. Hazard ratios (HRs) and 95% confidence intervals (CIs) obtained from univariate and multivariable-adjusted Cox proportional hazard models were used to assess the relationship between cancer mortality and kidney function while controlling for baseline variables of age, race, ethnicity, smoking status, diabetes, history of coronary heart disease, body mass index, systolic and diastolic blood pressure, total cholesterol, glucose, estrogen and aspirin therapy, and randomized antihypertensive group. HRs of all cancer mortality associated with level of eGFR were calculated in the unadjusted and multivariable-adjusted models. Estimated GFR was modeled as a continuous and categorical by eGFR groups: (≥ 90, 60 – 89.9, 45 – 59.9, < 45 mL/min/1.73 m^2^) variable. Given a relatively small sample size in the GFR < 30 mL/min/1.73 m^2^ strata, GFR < 45 mL/min/1.73 m^2^ was used as a cutoff point. All variables with an association with cancer at p < 0.05 in the unadjusted analyses were included in the multivariable adjusted analyses. However, smoking status was kept in all models since smoking is a well-established independent risk factor for cancer. To recognize potential effect modification, we used two-way interaction terms between the study predictor variables (eGFR) and all other covariates. Prespecified subgroups for these analyses included: 1)  men vs. women, 2) participants < 65 vs. ≥ 65 years, 3) Black vs. non-Black participants, 4) diabetic vs. nondiabetic participants, and 5) the presence or absence of CHD at baseline. To explore nonlinear relationships between CKD and cancer, we created adjusted spline models comparing each decile of GFR with the highest decile. For survival analyses, the follow-up period was defined from the time of study randomization (1994 – 1998) to time of cancer mortality. The follow-up period included both the randomized trial (mean follow-up duration 4.9 years) and subsequent extension-period follow-up (4 years). A p-value < 0.05 was used to indicate statistical significance for the results. However, given the many proportional hazard, subgroup, and interaction analyses performed, statistical significance at the 0.05 level should be interpreted cautiously. 

We also examined physician-reported diagnosis of new-incident cancer or death due to cancer during the clinical trial as an outcome; principles of analyses were similar, as detailed above. 

## Results 

Baseline characteristics of the ALLHAT study participants included in this analysis are shown in Table 1, overall and stratified by eGFR. There were several differences in baseline characteristics between participants by eGFR: participants with lower eGFR were older, more likely to be female, have baseline CHD, and were less likely to be Black. There were no statistically-significant differences between groups with regard to randomization to antihypertensive therapy arm. 

**Figure 2.**
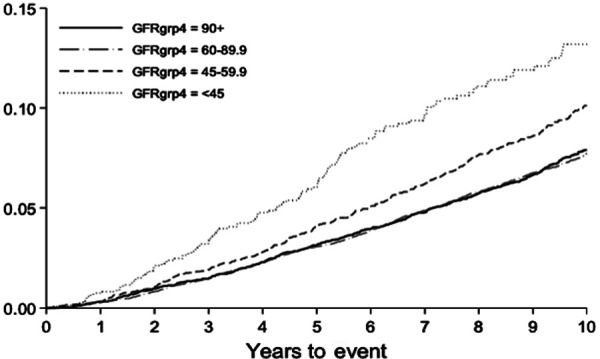
Cancer mortality (in-trial and post-trial combined) stratified by baseline eGFR.

During the trial and post-trial follow-up period, through December 31, 2006, 2,338 study participants had cancer-related mortality. Detailed site-specific cancer mortality stratified by eGFR is shown in Supplemental Table 1. Rates of cancer mortality were highest among patients with lower GFR; the 10-year cancer mortality rates were 7.90, 7.71, 10.11, and 13.19 per 100 persons in the eGFR ≥ 90, 60 – 89.9, 45 – 59.9, and < 45 mL/min/1.73 m^2^ groups, respectively (Table 2). The risk for cancer mortality was progressively higher in patients with strata of lower GFR (p for trend < 0.004) (Table 3) (Figure 2). Participants with an eGFR < 45 mL/min/1.73 m^2^ had the highest adjusted risk of cancer mortality compared to those with an eGFR ≥ 90 (adjusted HR 1.54 (95% CI 1.22 – 1.94). This was consistent for colon cancer (adjusted HR 2.28 (95% CI 1.12 – 4.62)), urinary-tract cancer (adjusted HR 2.95 (95% CI 1.14 – 7.65)) and “Other” cancers (adjusted HR 1.70 (95% CI 1.17 – 2.46)). 

**Figure 3.**
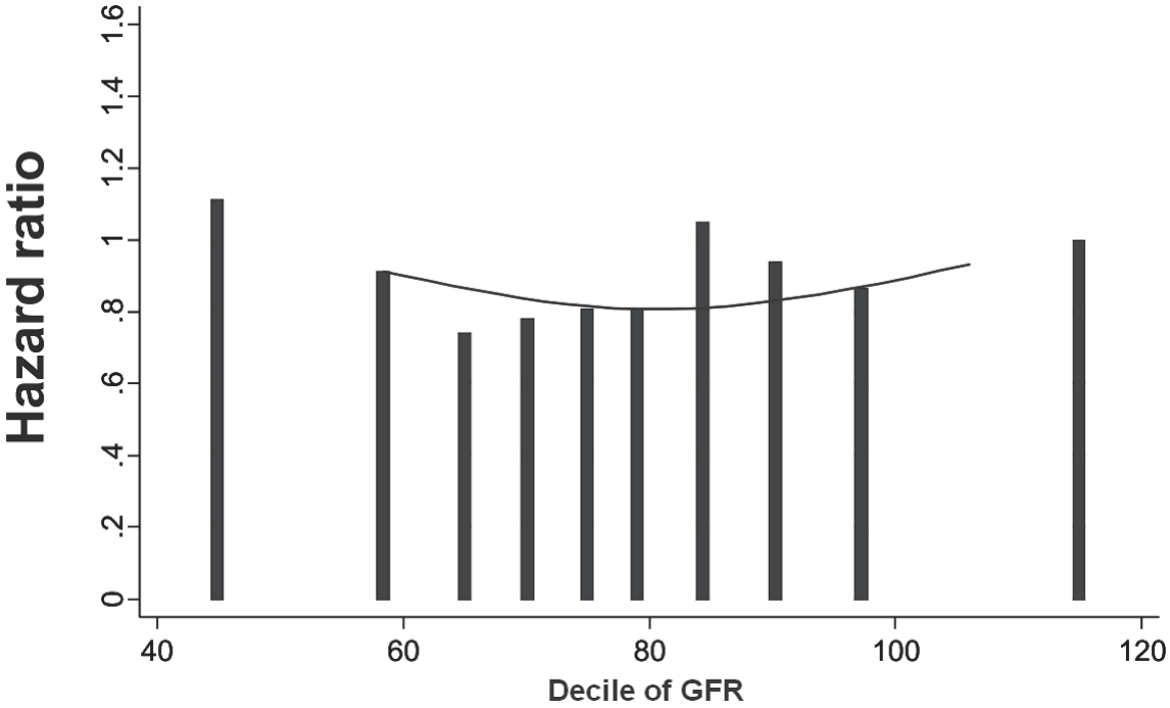
Spline analyses for hazard ratio for cancer mortality (in-trial and post-trial combined) by decile of eGFR) (each decile compared to the highest decile of GFR as the referent group).

When examined as a continuous variable in the adjusted model, eGFR was not independently associated with cancer mortality for all cancers (HR 1.00 (95% CI 0.98 – 1.03)), or for site-specific cancers (Supplemental Table 2). This was consistent across the subgroups of age, gender, diabetes, race, and CHD (Supplemental Table 3.) However, in the adjusted spline model, there appeared to be a nonlinear, U-shaped relationship between decline of eGFR and risk for cancer mortality; either very high or very low eGFR was associated with higher risk of cancer mortality (Figure 3). In regression models accounting for competing risk, the risk of cancer mortality in the GFR < 45 compared to the GFR > 90 stratum was attenuated (1.21 (0.96 – 1.54) (Supplemental Table 4). 

A physician-reported diagnosis of new-incident cancer or death due to cancer during the clinical trial phase occurred in 2,529 participants; rates were higher in participants with lower eGFR, with a graded increase in rates across strata of eGFR (Supplemental Table 5, Supplemental Figure 1). The risk for incident cancer/mortality during the in-trial period was higher in patients with lower eGFR strata (Supplemental Table 6). Following adjustment for potential confounders, participants with an eGFR < 45 mL/min/1.73 m^2^ had a 43% higher risk for incident cancer/mortality compared to those with GFR ≥ 90 mL/min/1.73 m^2^ (HR 1.43 (95% CI 1.13 – 1.79)). When eGFR was evaluated as a continuous variable in the adjusted model, the risk of incident cancer/cancer mortality was 2% higher for every 10 mL/min/1.73 m^2^ decrease in eGFR (Supplemental Table 7). This association was consistent across the subgroups of gender, diabetes, race, and CHD. There was a significant interaction by age (p = 0.03); every 10 mL/min/1.73 m^2^ decrease in eGFR was associated with a 4% higher risk of incident cancer/cancer mortality in participants above the age of 65 years, but not in those under the age of 65 years. 

## Discussion 

In this large cohort of hypertensive participants, we demonstrate that participants with an eGFR < 45 mL/min/1.73 m^2^ were at significantly higher risk of cancer mortality when compared to participants with eGFR > 90 mL/min/1.73 m^2^. This is particularly seen in increased risk of colon, urinary-tract, and “other” cancers but not lung cancer, breast cancer, or hematopoietic malignancy. These findings were generally consistent across subgroups defined by age, race, gender, diabetes, and pre-existing coronary heart disease. 

There has recently been considerable interest in studying the association between CKD and cancer, and cancer has been shown to be a leading cause of death in patients with CKD [13]. However, many studies have been relatively small, with selected populations, and, perhaps not surprisingly, have given conflicting results. In a prospective Australian study, men, but not women, with at least CKD stage 3 had a significantly increased risk for cancer with an eGFR < 55 mL/min/1.73 m^2^ [15]. Similarly, a Swedish cohort with a limited number of patients with CKD showed an association between kidney function and cancer, but only in young men [23]. Other studies of CKD patients with diabetes showed no association with cancer [17]. A more recent study showed increased cancer mortality (HR 1.91 (95% CI 1.32 – 2.76)) when comparing those with eGFR < 45 mL/min/1.73 m^2^ to those with eGFR > 75 mL/min/1.73 m^2^ [24]. A study of patients in the Kaiser healthcare system (Kaiser Permanente Northern California, a large integrated health care delivery system with a large population, representative of the local population) showed an increased incidence of renal-cell cancer among patients starting at eGFR < 60 mL/min/1.73 m^2^ compared to normal renal function; however, there was no association between CKD and all cancers [14]. Our study makes an important contribution to this field; it shows an independent association between CKD stages and cancer mortality in a large, multiethnic cohort. Our findings suggest that the presence of eGFR < 45 mL/min/1.73 m^2^ is associated with a higher risk of cancer-related mortality compared to eGFR ≥ 90 mL/min/1.73 m^2^. Most studies have only shown an association based on low vs. high eGFR or an association with a specific malignancy. The large sample size and distribution of eGFR in the ALLHAT population allowed a more refined estimation of the level of eGFR that is associated with increased cancer risk. In addition, the analysis of site-specific cancers also moves the field forward by documenting an increased risk of GI and urinary-tract cancers in patients with CKD. These observations are consistent with reports from previous studies, which showed increased GI and urinary-tract cancer [14, 18, 24, 25, 26]. Interestingly, there was no association of eGFR with lung and breast cancers, two of the most common cancers in the United States. 

The large sample size of the ALLHAT cohort allowed for meaningful subgroup analyses. Other recent cohort studies with a large sample size have primarily included Caucasian patients [14, 24]. In contrast, the ALLHAT sample was more diverse, with 34% of participants being Blacks and 19% Hispanic; an important finding of our study was that the association between CKD and cancer was consistent regardless of race/ethnicity, gender, or diabetes status. The finding that the association between eGFR and cancer was stronger in older patients is thought provoking and supports the need for further study in older patients. It is possible that initiation of dialysis or transplantation influenced the risk of cancer; however, the number of participants who reached ESRD was small [20, 21]. 

The association between CKD and cancer in this study may be partially explained by coexistent risk factors. ALLHAT participants with reduced eGFR had several baseline characteristics that were different from those with preserved eGFR. For example, participants with lower eGFR were older (mean age 71 years) compared to those with preserved eGFR (mean age 63 years). However, while adjustment for age and other risk factors attenuated the relationship between CKD and cancer, the association remained statistically significant. The association of eGFR with cancer mortality may not be linear, as shown in the spline analyses; this may reflect increased risk of cancer in patients who are chronically ill or frail but have an elevated eGFR in settings of poor muscle mass. Finally, the attenuation of the risk associated with CKD and cancer in competing risk models reinforces the complex relationship between multiple clinical outcomes in older patients with CKD. However, it is thought that etiologic associations are better characterized by traditional regression models rather than competing risk models [27]. The association of GFR with physician-diagnosed cancer or cancer mortality during the course of the trial was consistent with cancer mortality data, in showing a higher risk with lower GFR, although this did not achieve statistical significance. 

Although the pathophysiologic mechanisms mediating the relationship between CKD and cancer are not well established, possible links include lack of renal clearance of solutes, inflammation, and changes in the gut microbiome [28, 29, 30, 31, 32]. Our findings, along with other recent data, call for further research evaluating the mechanisms of association of CKD with cancer. 

Our study has several strengths: most notably it includes a large cohort of participants with a long period of follow-up. Additionally, the consistency of the results reported by physicians and those ascertained from national databases strengthens our conclusions about the association between CKD and incident cancer. The diversity of the study population with large numbers of diabetics, older patients, and Blacks also enhances the generalizability of the study findings. 

There are important limitations. The ALLHAT was not designed to evaluate the association between CKD and cancer, and therefore these findings are post-hoc and need confirmation. In addition, there was no direct way to ascertain prevalent cancer at baseline in ALLHAT participants. However, patients with diseases such as non-curable malignancies that were likely to lead to non-cardiovascular death over the course of the study were excluded from enrollment in the ALLHAT. It is thus unlikely that patients with undetected advanced cancers were enrolled in the study. All study participants had hypertension; extrapolation to patients without hypertension should be undertaken with caution. Additionally, participants with serum creatinine above 2.0 were excluded, thus limiting data from patients with advanced CKD. Patients with GFR less than 45 mL/min/1.73 m^2^ were more likely to be white and female. Whether this relates to the characteristics of the equation used to estimate GFR in unclear; in addition, although these differences were adjusted for in the analyses, the impact on the association between low eGFR and cancer is uncertain. It is possible that the limited precision and bias of the MDRD equation in estimating GFR, particularly at higher levels, may influence the associations seen in our study. 

In summary, this study demonstrates that eGFR < 45 (consistent with stage 3B CKD and below) is independently associated with an ~ 50% higher risk of cancer-related mortality compared to eGFR ≥ 90 mL/min/1.73 m^2^. These findings are generally consistent across subgroups of age, race, gender, and diabetes. Given the public-health burden of both CKD and cancer, these findings highlight the need for further research to understand the pathophysiologic mechanisms and strategies that may mediate this risk**.**

## Conflicts of interests 

Dr. Cushman has received honoraria from Takeda. Dr. Oparil has received honoraria from Daiichi Sankyo (Tokyo, Japan) and Novartis (Basel, Switherland). All other coauthors have no financial interests to disclose. Dr. Cutler is a contractor for the National Heart, Lung, and Blood Institute (NHLBI). The views expressed in this manuscript are those of the authors and do not necessarily represent those of the NHLBI. 

## Acknowledgments 

This study was supported by contract N01-HC-35130 and HHSN268201100036C from the US National Heart, Lung, and Blood Institute. The Institute’s role included involvement in design and conduct of the study; collection, management, analysis, and interpretation of the data; and preparation, review, and approval of the manuscript. The ALLHAT investigators acknowledge contributions of study medications supplied by Pfizer, Inc., (New York, NY, USA) (amlodipine and doxazosin), AstraZeneca (London, UK) (atenolol and lisinopril), and Bristol-Myers Squibb (New York, NY, USA) (pravastatin) and financial support provided by Pfizer, Inc. 

**Table 1.** Baseline characteristics of ALLHAT study participants followed for cancer mortality in (trial and post-trial combined) stratified by estimated glomerular filtration rate (eGFR).

	Estimated glomerular filtration rate (mL/min/1.73 m^2^)					
	Total	≥ 90	89.9 – 60	59.9 – 45	< 45	p-value
N*	31,349	8,027	17,778	4,293	1,251	
Age – mean years (SD)	66.9 (7.7)	63.3 (6.4)	67.3 (7.5)	70.5 (7.6)	71.7 (8.5)	< 0.001
Women – n (%)	14,561 (46.5)	3,752 (46.7)	7,905 (44.5)	2,205 (51.4)	699 (55.9)	< 0.001
Black – n (%)	10,990 (35.1)	4,042 (50.4)	5,438 (30.6)	1,107 (25.8)	403 (32.2)	< 0.001
Hispanic – n (%)	6,086 (19.4)	1,763 (22.0)	3,464 (19.5)	664 (15.5)	195 (15.6)	< 0.001
Education – mean years (SD)	11.0 (4.0)	10.9 (3.9)	11.1 (4.1)	10.9 (3.9)	10.4 (4.1)	< 0.001
Diabetes – n (%)	12,455 (41.6)	3,972 (51.2)	6,453 (38.1)	1,513 (37.4)	517 (43.7)	< 0.001
CHD – n (%)	7,965 (25.6)	1,682 (21.1)	4,619 (26.2)	1,292 (30.4)	372 (30.0)	< 0.001
ASCVD – n (%)	16,114 (51.4)	3,434 (42.8)	9,353 (52.6)	2,574 (60.0)	753 (60.2)	< 0.001
LVH on ECG – n (%)	5,160 (16.5)	1,315 (16.4)	2,912 (16.4)	714 (16.6)	219 (17.5)	0.752
Current or past smoking – n (%)	19,560 (62.4)	5,155 (64.2)	11,097 (62.4)	2,563 (59.7)	745 (59.6)	< 0.001
Aspirin – n (%)	11,320 (36.5)	2,558 (32.2)	6,631 (37.7)	1,657 (38.9)	474 (38.4)	< 0.001
Estrogen – in women, n (%)	2,590 (18.1)	729 (19.8)	1,439 (18.5)	349 (16.1)	73 (10.6)	< 0.001
Body mass index – mean (SD)	29.7 (6.1)	30.4 (6.5)	29.7 (6.0)	29.2 (5.8)	28.8 (6.2)	< 0.001
Blood pressure, mmHg – mean (SD)						
Systolic	146.2 (15.7)	145.9 (15.3)	146.3 (15.7)	146.5 (16.3)	147.0 (16.5)	0.054
Diastolic	84.0 (10.1)	84.9 (9.7)	84.1 (10.0)	82.7 (10.5)	82.0 (10.6)	< 0.001
Pulse, bpm – mean (SD)	73.5 (10.8)	74.7 (10.5)	73.1 (10.7)	72.9 (11.2)	74.0 (11.0)	< 0.001
Randomization arm						
Chlorthalidone – n (%)	14,370 (45.8)	3,606 (44.9)	8,199 (46.1)	1,999 (46.6)	566 (45.2)	0.225
Amlodipine – n (%)	8,492 (27.1)	2,245 (28.0)	4,768 (26.8)	1,156 (26.9)	323 (25.8)	0.181
Lisinopril – n (%)	8,487 (27.1)	2,176 (27.1)	4,811 (27.1)	1,138 (26.5)	362 (28.9)	0.407
Pravastatin (Lipid Trial) – n (%)	3,956 (12.6)	1,047 (13.0)	2,303 (13.0)	482 (11.2)	124 (9.9)	< 0.907
Fasting glucose, mg/dL – mean (SD)	123.3 (57.5)	137.3 (67.6)	118.9 (52.4)	115.2 (50.0)	122.3 (63.2)	< 0.001
Potassium, mEq/L – mean (SD)	4.3 (0.5)	4.2 (0.5)	4.3 (0.5)	4.4 (0.6)	4.5 (0.6)	< 0.001
Cholesterol, mg/dL – mean (SD)						
Total	216.0 (43.4)	214.8 (43.5)	215.2 (41.8)	219.3 (46.7)	224.1 (51.3)	< 0.001
LDL	135.8 (37.1)	134.7 (37.6)	135.7 (36.3)	137.0 (37.3)	140.9 (43.5)	< 0.001
HDL	46.8 (14.7)	48.3 (15.0)	46.5 (14.5)	45.9 (14.8)	45.1 (14.6)	< 0.001
Triglyceride, mg/dL – mean (SD)	175.9 (134.1)	169.9 (142.6)	173.4 (121.9)	189.3 (155.2)	202.9 (158.2)	< 0.001

*Variable total for education = 29,335; diabetes = 29,922; CHD = 31,108; smoking = 31,348; BMI = 31,253; pulse = 31,303; aspirin = 31,005; estrogen = 14,308; lipid trial = 7,873; glucose = 24,291; potassium = 31,230; cholesterol, total = 31,177, LDL = 29,261, HDL = 31,168, triglycerides = 31,165.

**Table 2.** Unadjusted event rates for cancer mortality (in-trial and post-trial combined) by eGFR and cancer type.

	Total	eGFR ≥ 90	eGFR (60 – 89.9)	eGFR (45 – 59.9)	eGFR < 45
	Number of events Event rate/100 (SE) 5-years 10-years	Number of events Event rate/100 (SE) 5-years 10-years	Number of events Event rate/100 (SE) 5-years 10-years	Number of events Event rate/100 (SE) 5-years 10-years	Number of events Event rate/100 (SE) 5-years 10-years
Cancer mortality	2,338 3.33 (0.10) 8.25 (0.17)	591 3.13 (0.20) 7.90 (0.33)	1,266 3.07 (0.13) 7.71 (0.22)	357 4.12 (0.32) 10.11 (0.54)	124 6.04 (0.73) 13.19 (1.20)
Prostate	191 0.51 (0.06) 1.29 (0.10)	40 0.48 (0.11) 1.09 (0.18)	104 0.40 (0.07) 1.12 (0.12)	36 0.82 (0.21) 2.30 (0.41)	11 1.75 (0.61) 2.70 (0.82)
Lung	719 1.10 (0.06) 2.61 (0.10)	191 1.09 (0.12) 2.59 (0.19)	389 0.96 (0.08) 2.46 (0.13)	110 1.62 (0.20) 3.08 (0.30)	29 1.35 (0.36) 3.45 (0.67)
Colon	230 0.33 (0.03) 0.87 (0.06)	54 0.25 (0.06) 0.73 (0.10)	132 0.35 (0.05) 0.90 (0.08)	31 0.33 (0.09) 0.92 (0.18)	13 0.65 (0.25) 1.29 (0.42)
Breast	107 0.28 (0.05) 0.81 (0.08)	29 0.30 (0.09) 0.82 (0.16)	50 0.24 (0.06) 0.68 (0.10)	21 0.40 (0.14) 1.25 (0.29)	7 0.32 (0.23) 1.06 (0.48)
Urinary-tract	110 0.15 (0.02) 0.39 (0.04)	23 0.08 (0.03) 0.32 (0.07)	59 0.14 (0.03) 0.34 (0.05)	18 0.22 (0.08) 0.55 (0.14)	10 0.56 (0.23) 1.12 (0.37)
Hematopoietic	179 0.25 (0.03) 0.64 (0.05)	39 0.20 (0.05) 0.52 (0.09)	104 0.25 (0.04) 0.66 (0.07)	30 0.36 (0.10) 0.78 (0.16)	6 0.30 (0.18) 0.67 (0.28)
Other	802 1.14 (0.06) 2.94 (0.11)	215 1.15 (0.12) 3.01 (0.21)	428 1.08 (0.08) 2.66 (0.13)	111 1.05 (0.16) 3.44 (0.34)	48 2.38 (0.47) 5.58 (0.86)

**Table 3.** Association between baseline eGFR and cancer mortality (in-trial and post-trial combined).

	eGFR (mL/min/1.73 m^2^) – HR (95% CI), p				
	≥ 90	60 – 89.9	45 – 59.9	< 45	Test for trend (p)
Cancer mortality					
Unadjusted	1.00 (ref)	0.98 (0.89 – 1.08)	1.27 (1.11 – 1.45)	1.88 (1.55 – 2.28)	< 0.001
Adjusted*	1.00 (ref)	0.91 (0.81 – 1.02)	1.09 (0.93 – 1.28)	1.54 (1.22 – 1.94)	0.004
Prostate					
Unadjusted	1.00 (ref)	1.12 (0.78 – 1.61)	2.08 (1.33 – 3.27)	3.01 (1.54 – 5.87)	< 0.001
Adjusted*	1.00 (ref)	1.07 (0.68 – 1.66)	1.70 (0.98 – 2.95)	1.83 (0.81 – 4.13)	0.028
Lung					
Unadjusted	1.00 (ref)	0.93 (0.79 – 1.11)	1.20 (0.95 – 1.52)	1.34 (0.91 – 1.98)	0.082
Adjusted*	1.00 (ref)	0.88 (0.71 – 1.09)	1.09 (0.82 – 1.46)	1.07 (0.66 – 1.73)	0.584
Colon					
Unadjusted	1.00 (ref)	1.12 (0.82 – 1.54)	1.21 (0.78 – 1.88)	2.15 (1.17 – 3.94)	0.048
Adjusted*	1.00 (ref)	1.29 (0.87 – 1.93)	0.90 (0.49 – 1.65)	2.28 (1.12 – 4.62)	0.238
Breast					
Unadjusted	1.00 (ref)	0.84 (0.53 – 1.33)	1.38 (0.79 – 2.42)	1.84 (0.81 – 4.21)	0.113
Adjusted**	1.00 (ref)	0.66 (0.39 – 1.13)	1.14 (0.58 – 2.23)	1.22 (0.45 – 3.35)	0.572
Urinary-tract					
Unadjusted	1.00 (ref)	1.17 (0.73 – 1.90)	1.65 (0.89 – 3.06)	3.99 (1.90 – 8.38)	0.001
Adjusted*	1.00 (ref)	0.99 (0.53 – 1.85)	1.54 (0.72 – 3.32)	2.95 (1.14 – 7.65)	0.023
Hematopoietic					
Unadjusted	1.00 (ref)	1.22 (0.85 – 1.77)	1.62 (1.01 – 2.61)	1.39 (0.59 – 3.30)	0.066
Adjusted*	1.00 (ref)	1.05 (0.65 – 1.70)	1.67 (0.92 – 3.01)	1.10 (0.37 – 3.24)	0.179
Other					
Unadjusted	1.00 (ref)	0.91 (0.78 – 1.08)	1.09 (0.86 – 1.36)	2.01 (1.47 – 2.75)	0.005
Adjusted*	1.00 (ref)	0.84 (0.69 – 1.03)	0.88 (0.66 – 1.17)	1.70 (1.17 – 2.46)	0.259

*Adjusted for age, gender (except for prostate cancer), race, ethnicity, smoking status, diabetes, history of coronary heart disease, body mass index, systolic and diastolic blood pressure, total cholesterol, glucose, aspirin use, and antihypertensive treatment arm. **Adjusted for age, race, ethnicity, smoking status, diabetes, history of coronary heart disease, body mass index, systolic and diastolic blood pressure, total cholesterol, glucose, history of estrogen therapy, aspirin use, and antihypertensive treatment arm.

## Supplemental material

Supplemental material Supplemental material
