# Supplementary material for: Association between chronic kidney disease and cancer mortality: A report from the ALLHAT
Source: Clin Nephrol. 2016 Nov 30;87(1):11–20. doi: 10.5414/CN108949 (PMC13055424; doi:10.5414/CN108949)
Supplement: Supplemental material [file clinnephrol-87-011-S01.pdf]

Clinical Nephrology, Vol. 87 – No. 1/2017 (S1-S5)

clinical nephrology
Online Supplement

©2017 Dustri-Verlag Dr. K. Feistle
ISSN 0301-0430

DOI 10.5414/CN108949
e-pub: November 30, 2016

Association between chronic kidney disease and cancer mortality: A report from the ALLHAT

Dhruti P. Chen[1], Barry R. Davis[2], Lara M. Simpson[2], William C. Cushman[3], Jeffrey A. Cutler[4], Mirela Dobre[5], Charles E. Ford[2], Gail T. Louis[6], Paul Muntner[2,7], Suzanne Oparil[8], Linda B. Piller[2], Sara L. Pressel[2], Mark J. Sarnak[9], Paul K. Whelton[6], Jackson T. Wright[5], and Mahboob Rahman[5], for the ALLHAT Collaborative Research Group

[1]Department of Medicine, Case Western Reserve University, University Hospitals Case Medical Center, Louis Stokes Cleveland Veterans Affairs Medical Center, Cleveland, OH, [2]Coordinating Center for Clinical Trials, Division of Biostatistics, University of Texas School of Public Health, Houston, TX, [3]Preventive Medicine Section, Memphis Veterans Affairs Medical Center, Memphis, TN, [4]National Heart, Lung and Blood Institute, Bethesda, MD, [5]Division of Nephrology and Hypertension, Case Western Reserve University, University Hospitals Case Medical Center, Louis Stokes Cleveland Veterans Affairs Medical Center, Cleveland, OH, [6]Tulane University Health Sciences Center, New Orleans, LA, [7]Department of Epidemiology at UAB, [8]Department of Medicine, University of Alabama at Birmingham, Birmingham, AL, and [9]Division of Nephrology, Tufts Medical Center, Boston, MA, USA

Online supplemental material

Data ascertainment

Of the 42,418 participants enrolled in ALLHAT, 10,522 were excluded from the analyses if they were assigned to doxazosin or had missing baseline serum creatinine data. The randomized groups were combined for the analyses reported here. Incident cancer was defined as the first fatal or nonfatal primary cancer diagnosed after study randomization during the ALLHAT clinical trial phase; the ascertainment of nonfatal cancer incidence was accomplished by utilizing clinician reports with documentation, and cancer mortality was on physician reports or National Death Index as described. Based upon physician report diagnoses or NDI*Plus* diagnoses for those lost to follow-up, incident cancer was classified by site of primary cancer, including prostate (in men), lung, colon, breast (in women), hyphenate urinary-tract (defined as kidney, ureter, ureterovesical junction, and bladder cancers), and hematopoietic "Other," a consolidation of anus, bone, brain, esophagus, gallbladder/biliary, liver, malignant fibrous histiocytoma, melanoma, mesothelioma, neuroendocrine, oral cavity, ovary, pancreas, salivary, sinus, small intestine, sarcoma, squamous cell, stomach, testicular/penile/scrotal, tongue, throat, thyroid, uterus, vagina/vulva, and unknown or unspecified primary diagnoses.

There are two sets of analyses. The supplemental set includes a composite endpoint of the first-occurring nonfatal or fatal clinic investigator report of organ-specific cancer from randomization through March 31, 2002. These reports are documented with pathology reports and/or death certificates. The categorical options of the ALLHAT Investigator Endpoint Form were mapped to International Classification of Disease-9 modification (ICD-9) for merging with post-trial data. The second set of analyses was of fatal cancer from randomization through December 31, 2006. If not identified by clinic investigator, cancer mortality was obtained from the National Death Index (NDI*Plus*)

Supplemental Table 1. Cancer mortality (in-trial and post-trial combined) by eGFR category and site.

		Estimated glomerular filtration rate (mL/min/1.73 m^2)			
Cancer	Total	≥ 90	89.9 – 60	59.9 – 45	< 45
Prostate	191	40	104	36	11
Lung	719	191	389	110	29
Colon	230	54	132	31	13
Breast	107	29	50	21	7
Urinary-tract*	110	23	59	18	10
Bone	4	1	2	1	0
Brain	38	7	21	6	4
DK (unspecified)	14	4	7	2	1
Esophagus	65	18	36	8	3
Gallbladder/biliary	25	7	15	2	1
Hematopoietic	179	39	104	30	6
Liver	71	26	32	11	2
Malignant fibrous histiocytoma	2	0	2	0	0
Melanoma	15	3	8	3	1
Mesothelioma	1	0	0	0	1
Oral cavity	15	6	7	1	1
Other	33	8	14	5	6
Ovary	29	4	20	5	0
Pancreas	144	39	82	16	7
Salivary	2	0	2	0	0
Sinus	4	1	3	0	0
Small intestine	4	2	2	0	0
Sarcoma	6	2	3	1	0
Squamous cell	2	0	1	1	0
Stomach	77	24	39	4	10
Testicular/penile/ scrotal	2	0	2	0	0
Tongue	7	2	4	1	0
Throat	35	11	16	5	3
Thyroid	4	1	3	0	0
Uterus	40	10	23	6	1
Vagina/vulva	1	1	0	0	0
UK primary	162	38	84	33	7
Total	2,338	591	1,266	357	124

*Urinary-tract includes: kidney, ureter, ureterovesical junction and bladder cancers.

Supplemental Table 2. Unadjusted and adjusted HR's for cancer mortality (in-trial and post-trial combined) using eGFR as a continuous variable.

Cancer type	HR per 10 unit decrease in eGFR
Cancer mortality	
Unadjusted	1.03 (1.01 – 1.05)
Adjusted*	1.00 (0.98 – 1.03)
Prostate	
Unadjusted	1.13 (1.04 – 1.22)
Adjusted*	1.04 (0.95 – 1.15)
Lung	
Unadjusted	1.00 (0.96 – 1.04)
Adjusted*	0.97 (0.93 – 1.02)
Colon	
Unadjusted	1.04 (0.97 – 1.11)
Adjusted*	1.03 (0.94 – 1.12)
Breast	
Unadjusted	1.00 (0.91 – 1.11)
Adjusted**	0.94 (0.83 – 1.05)
Urinary-tract	
Unadjusted	1.16 (1.05 – 1.29)
Adjusted*	1.12 (0.98 – 1.28)
Hematopoietic	
Unadjusted	1.07 (0.99 – 1.16)
Adjusted*	1.07 (0.96 – 1.19)
Other	
Unadjusted	1.02 (0.98 – 1.05)
Adjusted*	1.00 (0.95 – 1.04)

*Adjusted for baseline characteristics of age, gender (except for prostate cancer), race, ethnicity, smoking status, diabetes, history of coronary heart disease, body mass index, systolic and diastolic blood pressure, total cholesterol, glucose, aspirin use, and antihypertensive treatment arm. **Adjusted for baseline characteristics of age, race, ethnicity, smoking status, diabetes, history of coronary heart disease, body mass index, systolic and diastolic blood pressure, total cholesterol, glucose, estrogen therapy, aspirin use, and antihypertensive treatment arm.

database. Deaths identified from 1999 forward were obtained in ICD-10 form and were translated back to ICD-9. The organ-specific cancer mortality codes identified through NDI*Plus* were: prostate = 185, lung = 162, colon = 153 – 154.1, breast (in women) = 174, urinary-tract = 188 – 189, hematopoietic = 200 – 208, and “other” = 141 – 199, 235 – 239.

Supplemental Table 3. Subgroup analyses for the relationship between eGFR as a continuous variable (per 10 mL/min/1.73 m^2 decrease) and cancer mortality (in-trial and post-trial combined).

	Adjusted HR (95% CI)*	p-value	Interaction p-value
Overall	1.00 (0.98 – 1.03)	0.814	
Age 65+	1.04 (1.01 – 1.08)	**0.010**	0.144
Age < 65	0.98 (0.93 – 1.02)	0.315	
Women	1.01 (0.97 – 1.06)	0.577	0.868
Men	1.00 (0.96 – 1.03)	0.862	
Diabetes	0.98 (0.94 – 1.02)	0.265	0.753
No diabetes	1.02 (0.99 – 1.06)	0.224	
Black	1.01 (0.97 – 1.05)	0.584	0.475
Non-Black	1.00 (0.96 – 1.04)	0.975	
CHD	1.00 (1.00 – 1.01)	0.752	0.430
No CHD	1.00 (1.00 – 1.00)	0.628	

*Adjusted for age, gender, race, ethnicity, smoking status, diabetes, history of coronary heart disease, body mass index, systolic and diastolic blood pressure, total cholesterol, glucose, aspirin use, and antihypertensive treatment arm.

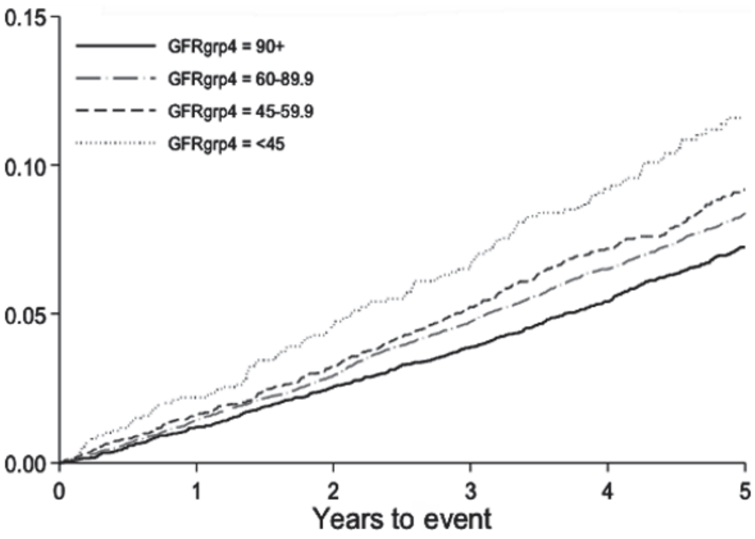

Supplemental Figure 1. Physician-reported cancer incidence or mortality during the clinical trial phase, stratified by eGFR.

Supplemental Table 4. Unadjusted and adjusted HR's for cancer mortality (in-trial and post-trial combined), analysis for using competing risk model.

eGFR (mL/min/1.73 m^2) – HR (95% CI), p				
	≥ 90	60 – 89.9	45 – 59.9	< 45
Cancer mortality				
Unadjusted	1.00 (ref)	0.98 (0.89 – 1.08)	1.27 (1.11 – 1.45)	1.88 (1.55 – 2.28)
Adjusted*	1.00 (ref)	0.91 (0.81 – 1.02)	1.09 (0.93 – 1.28)	1.54 (1.22 – 1.94)
Sub HR's for competing risk regression models				
Unadjusted	1.00 (ref)	0.96 (0.87 – 1.06)	1.12 (0.99 – 1.28)	1.37 (1.12 – 1.66)
Adjusted*	1.00 (ref)	0.92 (0.82 – 1.04)	1.03 (0.87 – 1.21)	1.21 (0.96 – 1.54)

*Adjusted for baseline characteristics of age, gender, race, ethnicity, smoking status, diabetes, history of coronary heart disease, body mass index, systolic and diastolic blood pressure, total cholesterol, glucose, aspirin use, and antihypertensive treatment arm.

Supplemental Table 5. Unadjusted event rates for physician-reported cancer incidence or mortality during the clinical trial phase by eGFR and cancer type.

Type of cancer	Total	eGFR ≥ 90	eGFR 89.9-60	eGFR 59.9-45	eGFR <45
	Number of events<br>5-year rate/100 (SE)	Number of events<br>5-year rate/100 (SE)	Number of events<br>5-year rate/100 (SE)	Number of events<br>5-year rate/100 (SE)	Number of events<br>5-year rate/100 (SE)
All	2529<br>8.31 (0.17)	565<br>7.24 (0.32)	1473<br>8.38 (0.23)	366<br>9.18 (0.49)	125<br>11.58 (1.06)
Prostate	554<br>3.42 (0.15)	112<br>2.50 (0.26)	356<br>3.76 (0.21)	64<br>3.44 (0.45)	22<br>4.63 (1.06)
Lung	468<br>1.51 (0.08)	117<br>1.36 (0.14)	258<br>1.48 (0.10)	74<br>1.88 (0.24)	19<br>1.65 (0.42)
Colon	313<br>1.03 (0.06)	58<br>0.81 (0.12)	194<br>1.09 (0.09)	48<br>1.12 (0.18)	13<br>1.27 (0.37)
Breast	252<br>1.80 (0.12)	59<br>1.60 (0.22)	141<br>1.86 (0.16)	42<br>1.97 (0.33)	10<br>1.50 (0.50)
Urinary-tract*	187<br>0.63 (0.05)	42<br>0.55 (0.09)	105<br>0.60 (0.07)	30<br>0.77 (0.15)	10<br>1.04 (0.34)
Hematopoietic	160<br>0.52 (0.05)	37<br>0.43 (0.08)	86<br>0.49 (0.06)	29<br>0.76 (0.16)	8<br>0.73 (0.30)
Other**	595<br>2.24 (0.09)	140<br>2.20 (0.18)	333<br>2.09 (0.12)	79<br>2.30 (0.26)	43<br>4.59 (0.73)

*Urinary-tract includes: kidney, ureter, ureterovesical junction and bladder cancers. **Other includes: anus, bone, brain, esophagus, gallbladder/biliary, liver, malignant fibrous histiocytoma, melanoma, mesothelioma, neuroendocrine, oral cavity, ovary, pancreas, salivary, sinus, small intestine, sarcoma, squamous cell, stomach, testicular/penile/scrotal, tongue, throat, thyroid, uterus, vagina/vulva, and unknown primary.

Supplemental Table 6. Unadjusted and adjusted event rates for physician-reported cancer incidence or mortality during the clinical trial phase, by eGFR and cancer type.

	eGFR (mL/min/1.73 m^2) – HR (95% CI), p					
Type of cancer	≥ 90	89.9 – 60	59.9 – 45	< 45	Test for trend (p)	HR per 10 unit decrease in eGFR
All						
Unadjusted	1.00 (ref)	1.17 (1.06 – 1.29)	1.24 (1.09 – 1.41)	1.66 (1.37 – 2.01)	< 0.001	1.04 (1.02 – 1.07)
Adjusted*	1.00 (ref)	1.05 (0.93 – 1.18)	1.09 (0.93 – 1.28)	1.43 (1.13 – 1.79)	0.094	1.02 (1.00 – 1.05)
Prostate						
Unadjusted	1.00 (ref)	1.43 (1.15 – 1.77)	1.25 (0.91 – 1.70)	1.82 (1.14 – 2.90)	0.010	1.06 (1.02 – 1.11)
Adjusted*	1.00 (ref)	1.13 (0.88 – 1.45)	1.05 (0.73 – 1.50)	1.36 (0.78 – 2.38)	0.420	1.02 (0.97 – 1.08)
Lung						
Unadjusted	1.00 (ref)	1.05 (0.83 – 1.32)	1.26 (0.93 – 1.71)	1.28 (0.78 – 2.11)	0.119	1.01 (0.97 – 1.06)
Adjusted*	1.00 (ref)	0.98 (0.74 – 1.28)	1.16 (0.80 – 1.67)	0.96 (0.52 – 1.79)	0.652	1.00 (0.94 – 1.06)
Colon						
Unadjusted	1.00 (ref)	1.50 (1.11 – 2.02)	1.57 (1.06 – 2.32)	1.74 (0.95 – 3.18)	0.010	1.07 (1.01 – 1.14)
Adjusted*	1.00 (ref)	1.47 (1.03 – 2.11)	1.28 (0.79 – 2.06)	1.55 (0.78 – 3.07)	0.231	1.04 (0.97 – 1.12)
Breast						
Unadjusted	1.00 (ref)	1.17 (0.85 – 1.59)	1.24 (0.83 – 1.86)	1.10 (0.56 – 2.15)	0.392	1.04 (0.98 – 1.11)
Adjusted**	1.00 (ref)	0.98 (0.68 – 1.43)	1.07 (0.66 – 1.74)	0.88 (0.40 – 1.93)	0.995	1.01 (0.93 – 1.10)
Urinary-tract						
Unadjusted	1.00 (ref)	1.08 (0.75 – 1.54)	1.32 (0.82 – 2.12)	1.77 (0.89 – 3.54)	0.088	1.10 (1.02 – 1.19)
Adjusted*	1.00 (ref)	1.01 (0.65 – 1.55)	1.11 (0.61 – 2.01)	2.10 (0.97 – 4.56)	0.173	1.11 (1.00 – 1.22)
Hematopoietic						
Unadjusted	1.00 (ref)	1.18 (0.78 – 1.77)	1.61 (0.97 – 2.69)	1.41 (0.59 – 3.37)	0.089	1.07 (0.99 – 1.17)
Adjusted*	1.00 (ref)	1.01 (0.58 – 1.73)	1.41 (0.72 – 2.76)	1.62 (0.62 – 4.24)	0.174	1.08 (0.96 – 1.21)
Other						
Unadjusted	1.00 (ref)	0.98 (0.82 – 1.18)	1.04 (0.81 – 1.35)	2.12 (1.53 – 2.93)	0.004	1.03 (0.99 – 1.07)
Adjusted*	1.00 (ref)	0.93 (0.74 – 1.16)	0.92 (0.67 – 1.26)	1.74 (1.17 – 2.59)	0.140	1.00 (0.95 – 1.05)

*Adjusted for baseline characteristics of age, gender (except for prostate cancer), race, ethnicity, smoking status, diabetes, history of coronary heart disease, body mass index, systolic and diastolic blood pressure, total cholesterol, glucose, aspirin use, and antihypertensive treatment arm. **Adjusted for baseline characteristics of age, race, ethnicity, smoking status, diabetes, history of coronary heart disease, body mass index, systolic and diastolic blood pressure, total cholesterol, glucose, estrogen therapy, aspirin use, and antihypertensive treatment arm.

Supplemental Table 7. Subgroup analyses for the relationship between estimated glomerular filtration rate as a continuous variable (per 10 mL/min/1.73 m^2 decrease) and physician-reported cancer incidence or mortality during the clinical trial phase.

	Adjusted HR (95% CI)*	p-value	Interaction p-value
Overall	1.02 (1.00 – 1.05)	0.094	
Age 65+	1.04 (1.00 – 1.07)	**0.027**	**0.025**
Age < 65	1.02 (0.98 – 1.06)	0.362	
Women	1.02 (0.98 – 1.07)	0.335	0.936
Men	1.02 (0.99 – 1.05)	0.188	
Diabetes	1.01 (0.97 – 1.05)	0.540	0.999
No diabetes	1.03 (0.99 – 1.06)	0.109	
Black	1.05 (1.01 – 1.09)	**0.011**	0.065
Non-Black	1.00 (0.97 – 1.04)	0.858	
CHD	1.00 (0.99 – 1.00)	0.737	0.213
No CHD	1.00 (0.99 – 1.00)	0.070	

*Adjusted for age, gender, race, ethnicity, smoking status, diabetes, history of coronary heart disease, body mass index, systolic and diastolic blood pressure, total cholesterol, glucose, aspirin use, and antihypertensive treatment arm.
